# Supplementary material for: Empirical methods for controlling false positives and estimating confidence in ChIP-Seq peaks
Source: BMC Bioinformatics. 2008 Dec 5;9:523. doi: 10.1186/1471-2105-9-523 (PMC2628906; doi:10.1186/1471-2105-9-523)
Supplement: Additional File 1 — A variety of html documents from the USeq web site detailing the available applications, their best usage, output file type descriptions, command line menus, etc. [file 1471-2105-9-523-S1.zip › USeqUserGuides/JavaDoc.zip!/JavaDoc/trans/anno/Region.html]

Region


|  |  |  |
| --- | --- | --- |
| **Overview** \| **Package** \| **Class** \| **Use** \| **Tree** \| **Deprecated** \| **Index** \| **Help** | |  |
| **PREV CLASS**   **NEXT CLASS** | **FRAMES**    **NO FRAMES**     **All Classes** |  |
| SUMMARY: NESTED \| FIELD \| CONSTR \| METHOD | DETAIL: FIELD \| CONSTR \| METHOD |  |


---


## trans.anno Class Region

```
java.lang.Object
  trans.anno.Region
```

**All Implemented Interfaces:**: java.io.Serializable

---

``` public class Region extends java.lang.Object implements java.io.Serializable ```

Represents a genomic region. See Coordinate for a more simple class.

**See Also:**: Serialized Form

---

**Constructor Summary**

`Region(java.lang.String chromosome,
int start,
int end,
java.lang.String notes)`


| **Method Summary** | |
| --- | --- |
| `int` | `bpIntersectionSameChromosome(Region other)`             Assumes regions are on the same chromosome, and the end base is included, not interbase numbering. |
| `double` | `calculateGCContent()`             Returns the fraction of GC provided the Region.gcContent[] is loaded. |
| `java.lang.String` | `getChromosome()` |
| `int` | `getEnd()` |
| `boolean[]` | `getGcContent()` |
| `java.util.HashSet` | `getHits()` |
| `int` | `getLength()`             Returns end - start +1, thus last base is included, not interbase numbering! |
| `java.lang.String` | `getNotes()` |
| `int` | `getStart()` |
| `boolean` | `intersect(Interval interval)` |
| `boolean` | `intersect(TPMapLine line)` |
| `boolean` | `intersect(Window window, int sizeOligo, double fractionAcceptibleCoverage)`             Returns true only if region contains or is contained or is covered by >= fractionAcceptibleCoverage by a window. |
| `static Region[]` | `loadBinaryCoordinates(java.io.File file)`             Reads binary chrom start stop file into Region[] |
| `static Region[]` | `loadWriteBinaryRegions(java.io.File regionsFile)`             Looks for a binary version of the file xxx.corr, if found loads binary, otherwise it parses the txt file and then writes the binary for future use. |
| `int` | `overlap(Interval interval)`             Returns -1 for no overlap, or a positive int for the # bases of overlap. |
| `static Region[]` | `parseRegions(java.io.File file)`             Parses a file for a tab delimited list of at minimum, chrom, start, stop, (optional) notes into a GenomicRegion[]. |
| `void` | `setChromosome(java.lang.String chromosome)` |
| `void` | `setEnd(int end)` |
| `void` | `setGcContent(boolean[] gcContent)` |
| `void` | `setHits(java.util.HashSet hits)` |
| `void` | `setNotes(java.lang.String notes)` |
| `void` | `setStart(int start)` |
| `static java.util.HashMap` | `splitByChromosome(Region[] sorted)`             Given a chromosome SORTED array of Regions returns a HashMap containing chromosome: sorted chromo specific Region[] |
| `java.lang.String` | `toString()` |
| `static void` | `writeBinaryCoordinates(java.io.File file, Region[] r)`             Writes the chrom start stop of a Region[] as a binary file. |

**Methods inherited from class java.lang.Object**

`equals, getClass, hashCode, notify, notifyAll, wait, wait, wait`

**Constructor Detail**

### Region

```
public Region(java.lang.String chromosome,
              int start,
              int end,
              java.lang.String notes)
```


**Method Detail**

### getLength

```
public int getLength()
```

:   Returns end - start +1, thus last base is included, not interbase numbering!

---


### loadWriteBinaryRegions

```
public static Region[] loadWriteBinaryRegions(java.io.File regionsFile)
```

:   Looks for a binary version of the file xxx.corr, if found loads binary, otherwise it parses the txt file
    and then writes the binary for future use.

---


### loadBinaryCoordinates

```
public static Region[] loadBinaryCoordinates(java.io.File file)
```

:   Reads binary chrom start stop file into Region[]

---


### writeBinaryCoordinates

```
public static void writeBinaryCoordinates(java.io.File file,
                                          Region[] r)
```

:   Writes the chrom start stop of a Region[] as a binary file.

---


### splitByChromosome

```
public static java.util.HashMap splitByChromosome(Region[] sorted)
```

:   Given a chromosome SORTED array of Regions returns a HashMap
    containing chromosome: sorted chromo specific Region[]

---


### toString

```
public java.lang.String toString()
```

:   **Overrides:**: `toString` in class `java.lang.Object`

---


### calculateGCContent

```
public double calculateGCContent()
```

:   Returns the fraction of GC provided the Region.gcContent[] is loaded.
    Otherwise returns zero.

---


### intersect

```
public boolean intersect(Interval interval)
```

---


### intersect

```
public boolean intersect(TPMapLine line)
```

---


### intersect

```
public boolean intersect(Window window,
                         int sizeOligo,
                         double fractionAcceptibleCoverage)
```

:   Returns true only if region contains or is contained or is covered by >= fractionAcceptibleCoverage by a window.
    ie 0.5 for 50% or region covered by a window.

---


### bpIntersectionSameChromosome

```
public int bpIntersectionSameChromosome(Region other)
```

:   Assumes regions are on the same chromosome, and the end base is included, not interbase numbering.
    Returns 0 if the regions abut, positive ints for each base in the gap (ie 1= 1bp between an end of 12 and a start of 14),
    negative ints for each base of intersection (ie -1= 1bp overlap),
    max negative int is the length of the smaller region (ie end - start +1).

---


### overlap

```
public int overlap(Interval interval)
```

:   Returns -1 for no overlap, or a positive int for the # bases of overlap.
    Doesn't check chromosome!

---


### parseRegions

```
public static Region[] parseRegions(java.io.File file)
```

:   Parses a file for a tab delimited list of at minimum, chrom, start, stop, (optional) notes into a
    GenomicRegion[].

---


### getChromosome

```
public java.lang.String getChromosome()
```

---


### setChromosome

```
public void setChromosome(java.lang.String chromosome)
```

---


### getEnd

```
public int getEnd()
```

---


### setEnd

```
public void setEnd(int end)
```

---


### getHits

```
public java.util.HashSet getHits()
```

---


### setHits

```
public void setHits(java.util.HashSet hits)
```

---


### getNotes

```
public java.lang.String getNotes()
```

---


### setNotes

```
public void setNotes(java.lang.String notes)
```

---


### getStart

```
public int getStart()
```

---


### setStart

```
public void setStart(int start)
```

---


### getGcContent

```
public boolean[] getGcContent()
```

---


### setGcContent

```
public void setGcContent(boolean[] gcContent)
```


---


|  |  |  |
| --- | --- | --- |
| **Overview** \| **Package** \| **Class** \| **Use** \| **Tree** \| **Deprecated** \| **Index** \| **Help** | |  |
| **PREV CLASS**   **NEXT CLASS** | **FRAMES**    **NO FRAMES**     **All Classes** |  |
| SUMMARY: NESTED \| FIELD \| CONSTR \| METHOD | DETAIL: FIELD \| CONSTR \| METHOD |  |


---
